# Selective photodissociation of tailored molecular tags as a tool for quantum optics

**DOI:** 10.3762/bjnano.8.35

**Published:** 2017-02-02

**Authors:** Ugur Sezer, Philipp Geyer, Moritz Kriegleder, Maxime Debiossac, Armin Shayeghi, Markus Arndt, Lukas Felix, Marcel Mayor

**Affiliations:** 1 Faculty of Physics, VCQ, University of Vienna, Boltzmanngasse 5, A-1090 Vienna, Austria; 2 Department of Chemistry, University of Basel, St. Johannsring 19, CH-4056 Basel, Switzerland; 3 Institute of Nanotechnology (INT), Karlsruhe Institute of Technology (KIT), Hermann-von-Helmholtz-Platz 1, D-76344 Eggenstein-Leopoldshafen, Germany; 4 Lehn Institute of Functional Materials (LIFM), Sun Yat-Sen University (SYSU), Xingang Rd. W., Guangzhou, China

**Keywords:** molecular quantum optics, photodepletion, photodissociation, synthetic photo-tags

## Abstract

Recent progress in synthetic chemistry and molecular quantum optics has enabled demonstrations of the quantum mechanical wave–particle duality for complex particles, with masses exceeding 10 kDa. Future experiments with even larger objects will require new optical preparation and manipulation methods that shall profit from the possibility to cleave a well-defined molecular tag from a larger parent molecule. Here we present the design and synthesis of two model compounds as well as evidence for the photoinduced beam depletion in high vacuum in one case.

## Introduction

Chemistry builds on the fact that the electronic structure, dynamics and properties of molecules are determined or influenced by quantum effects. However, it has only recently been experimentally verified that also the translational motion of an entire complex molecule has to be described by a quantum wave function under appropriate circumstances [1–2].

Since the de Broglie wave nature of atoms has already become a central element in sensitive matter-wave interferometers with applications in geodesy, navigation and fundamental science [3], it is now natural to ask how complex an object may be while still displaying quantum delocalization over macroscopic times and length scales [4–5]. Matter-waves can then become tools for chemistry too, for instance in novel measurements of molecular properties [6–8].

First experiments with complex molecules realized the idea of Young’s double slit experiment and demonstrated diffraction of fullerenes [1] and functionalized phthalocyanines [9] at nanomechanical gratings. Later studies used variants of Talbot–Lau interferometry to demonstrate the quantum wave nature of more than a dozen of different molecules and clusters [10–16], up to tailored porphyrin derivatives as massive as 10 kDa [17]. In these experiments, every single molecule contained 810 atoms and yet it still needed to be described by a quantum wave function with a de Broglie wavelength of 300–500 fm. The molecular coherence spanned several hundred nanometers for several milliseconds [17].

We are now looking for new techniques which shall enable new molecular matter-wave experiments. We are in particular interested in identifying an optical depletion mechanism for molecular beams, since this will enable the realization of absorptive optical gratings. Standing waves of light can imprint a spatially periodic structure onto molecular beams – very similar to nanomechanical material gratings – if they are capable of depleting the molecular beam in the antinodes of the optical grating. In previous studies, this goal has been achieved by exposing molecules to a vacuum ultra-violet (VUV) light emitted by a laser of 157.6 nm wavelength [18–19]. The light field can ionize or dissociate the incident clusters of molecules in the anti-nodes of the optical gratings [13,18,20].

These previous attempts proved successful but were not yet generally scalable to covalently bound organic molecules. In particular, large biomolecules – which are interesting candidates for quantum-interference experiments and gas-phase metrology [7] – often neither ionize nor dissociate upon absorption of a single photon, not even at 7.9 eV photon energy [21].

Here we address this challenge and study tailored tags that are optimized to respond to light of lower photon energy with cleavage at a deterministic location. Click chemistry shall then allow attaching them to a wide class of analyte molecules and thus solve the problem.

## Results

### Design of the model compounds

**Figure 1:**
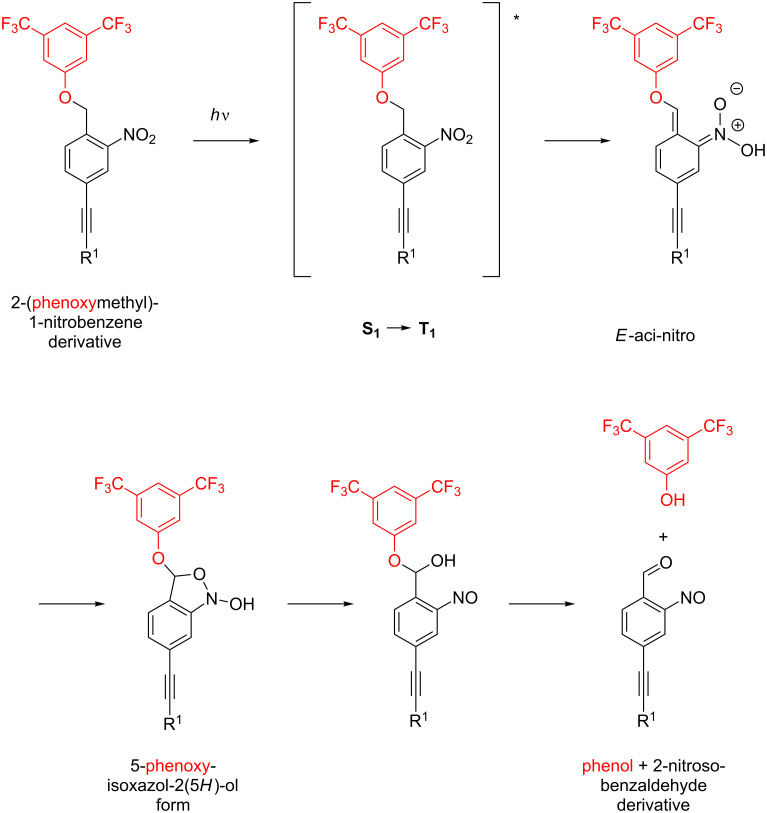
Sketch of the photocleavable 2-(phenoxymethyl)-1-nitrobenzene subunit and its intramolecular photon induced degradation sequence which leads to the release of the phenol subunit (red).

A large number of photocleavable groups can be found in the literature [22]. Here, we have chosen a 2-(phenoxymethyl)-1-nitrobenzene motive as photocleavable linker, because of its photoinduced cleavage sequence, sketched in Figure 1. It requires only the intramolecular exchange of electrons and atoms but no solvent molecule in this cleaving mechanism. Our hypothesis is that this independence increases the cleavage probability also for molecules in high vacuum – even though the solvent may still support the process by stabilizing certain intermediates. The photocleavable label was further decorated with an ethynyl group in 5-position as a functional group that enables various coupling procedures and which can link the functional subunit to the structure of interest (e.g., R^1^ in Figure 1). We also add CF_3_ groups to the phenol subunit which shall be released upon photocleavage in order to increase the molecular weight while keeping the molecular polarizability low. Former experiments have shown that the perfluoroalkyl-functionalization of complex molecules can reduce the intermolecular adhesion, facilitate the volatilization and thus also facilitate the formation of a molecular beam [23–24]. It is also important to tailor the mass of the ejected fragment because one may later use the momentum transfer in this process to remove both fragments from an initially tightly collimated molecular beam.

The photoresponse of the nitrobenzyl group has been studied in great detail in solution [22,25] and the sequence resulting in the separation of both subunits is summarized in Figure 1. After excitation with UV-light from its equilibrium form to the singlet state **S_1_**, the molecule decays to the triplet state **T_1_** which can undergo a hydrogen transfer to yield the *E*-aci-nitro form. The latter decays over the cyclic isoxazol form to a 2-nitrosobenzaldehyde and a phenol subunit (red in Figure 1). Even though the reaction sequence is well understood in solution, it remains an open question if a similar cleavage occurs and which decay channels are accessible when the photoactive molecule is isolated in high vacuum at 300 K or more. For that to happen, a number of complex reactions and atomic position changes need to be allowed. They may change in the absence of solvent molecules which provide both a polarizable environment, possibly stabilizing dipole moments of transition states, and a thermal bath for energy and momentum exchange.

For our proof-of-principle investigation we assembled the model compound **1**, which consists of a central benzene core and three of the photocleavable subunits. The symmetric arrangement of three subunits was meant to lower the resonant absorption energy and to enhance the effective cross section for photoabsorption and cleavage.

**Scheme 1:**
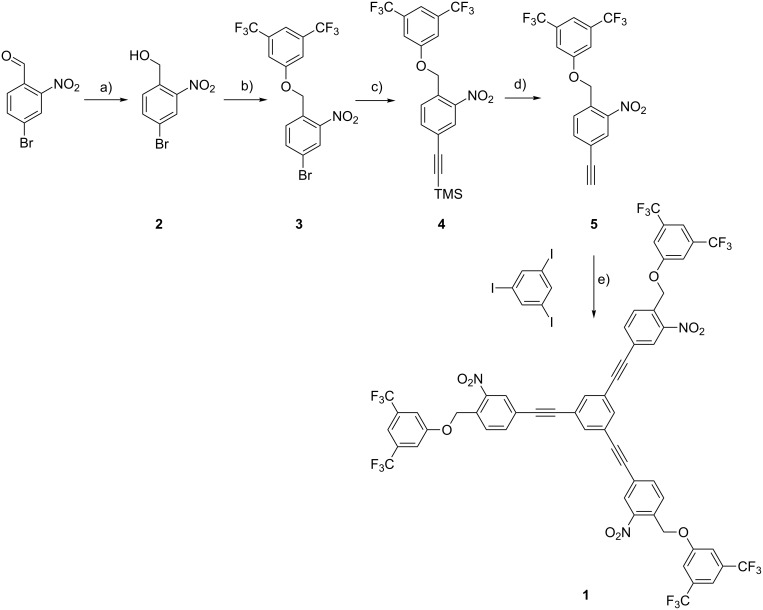
Synthesis of the trimeric target structure **1**. Reagents and conditions: a) NaBH_4_, THF, 30 min, rt, quant.; b) 3,5-bis(trifluoromethyl)phenol, DIAD, Ph_3_P, THF, 12 h, rt, 96%; c) TMS-CC-H, Pd(PPh_3_)_4_, CuI, TEA, 90 °C, 12 h, 99%; d) TBAF (1 M in THF), CH_2_Cl_2_, rt, 30 min, quant.; e) 1,3,5-triiodobenzene, **5** (4.5 equiv), Pd(PPh_3_)_4_, CuI, THF/TEA (3:1), 12 h, 50 °C, 80%.

The synthesis of the target structure **1** is displayed in Scheme 1. Reduction of the commercially available 4-bromo-2-nitrobenzaldehyde with sodium borohydride gave the benzyl alcohol **2**, which was obtained in quantitative yield after column chromatography (CC) as colourless solid. Applying Mitsunobu reaction conditions, the benzyl ether **3** was obtained from **2** and 3,5-bis(trifluoromethyl)phenol. The reagents were dissolved in 0 °C cold tetrahydrofuran (THF) while diisopropyl azodicarboxylate (DIAD) was added dropwise. After stirring for 12 h in the dark, work-up followed by CC provided **3** in 96% yield as colourless solid. The bromine substituent of **3** was substituted by trimethylsilylacetylene using Sonogashira conditions. The aryl bromide **3** and trimethylsilylacetylene were dissolved in triethylamine (TEA) and Pd(PPh_3_)_4_ and CuI were added as catalysts, before the reaction mixture was refluxed for 12 h. Work-up by CC provided the TMS-ethynyl functionalized benzyl ether **4** in 99% yield as colourless solid. Treatment of **4** with tetrabutylammonium fluoride (TBAF) in a dichloromethane/THF mixture provided the free arylethynyl **5** in quantitative yield. The trimeric target compound **1** was also synthesized using Sonogashira coupling conditions. 1,3,5-Triiodobenzene together with 4.5 equiv of the arylethynyl **5** were dissolved in a THF/TEA (3:1) mixture, to which catalytic amounts (5 mol %) of Pd(PPh_3_)_4_ and CuI were added before the reaction mixture was warmed up to 50 °C for 12 h. Aqueous work-up and recrystallization of the crude reaction product from chloroform provided the target compound **1** in 80% yield as colourless solid.

All new compounds were fully characterized by ^1^H, ^19^F and ^13^C NMR spectroscopy and high-resolution mass spectrometry (HRMS). Detailed experimental protocols together with analytical data are provided in Supporting Information File 1.

### Photocleavage in solution

**Figure 2:**
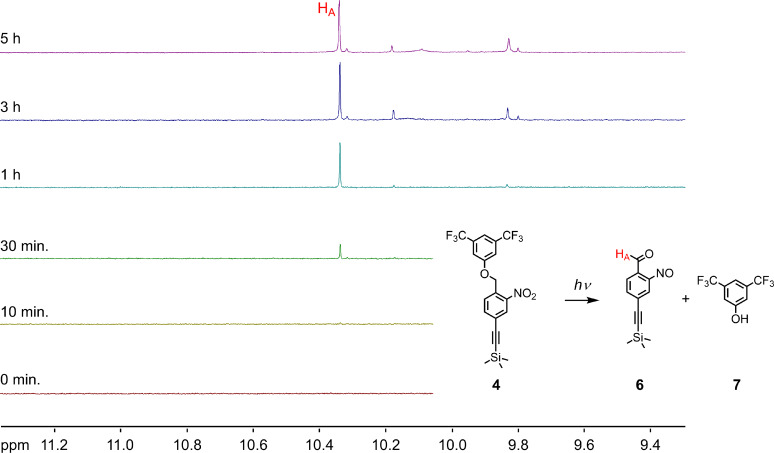
Qualitative photocleavage experiment of the monomer **4** irradiated at 355 nm inside the NMR spectrometer. The background displays the increase of the aldehyde signal of the 2-nitrosoaldehyde **6** with increasing light exposure.

The photoinduced degradation of the target compound was first studied in solution. Initially, we planned to investigate the photocleavage of the target trimer **1** by NMR spectroscopy to be able to analyse the products of the photodegradation. Due to the poor solubility of the trimer **1**, these studies were made with the monomeric building block **4**, which allowed for suitable concentrations in dichloromethane-*d*_2_ for the NMR experiment. In a qualitative degradation experiment, the NMR tube was directly irradiated inside the NMR spectrometer with a glass fibre exposing the sample to the 355 nm light from an 8 W UV lamp, filtered by a monochromator with a spectral transmission band of 2 nm. As expected, the formation of the nitrosoaldehyde **6** and the 3,5-bis(trifluoromethyl)phenol (**7**) were observed during irradiation. Figure 2 displays the increase of the aldehyde proton (H_A_) at 10.35 ppm with increasing duration of the light exposure.

The limited solubility of the trimer **1** prevented the investigation of its photodegradation by the same set-up. We therefore analysed its photoresponse using UV absorption spectroscopy.

**Figure 3:**
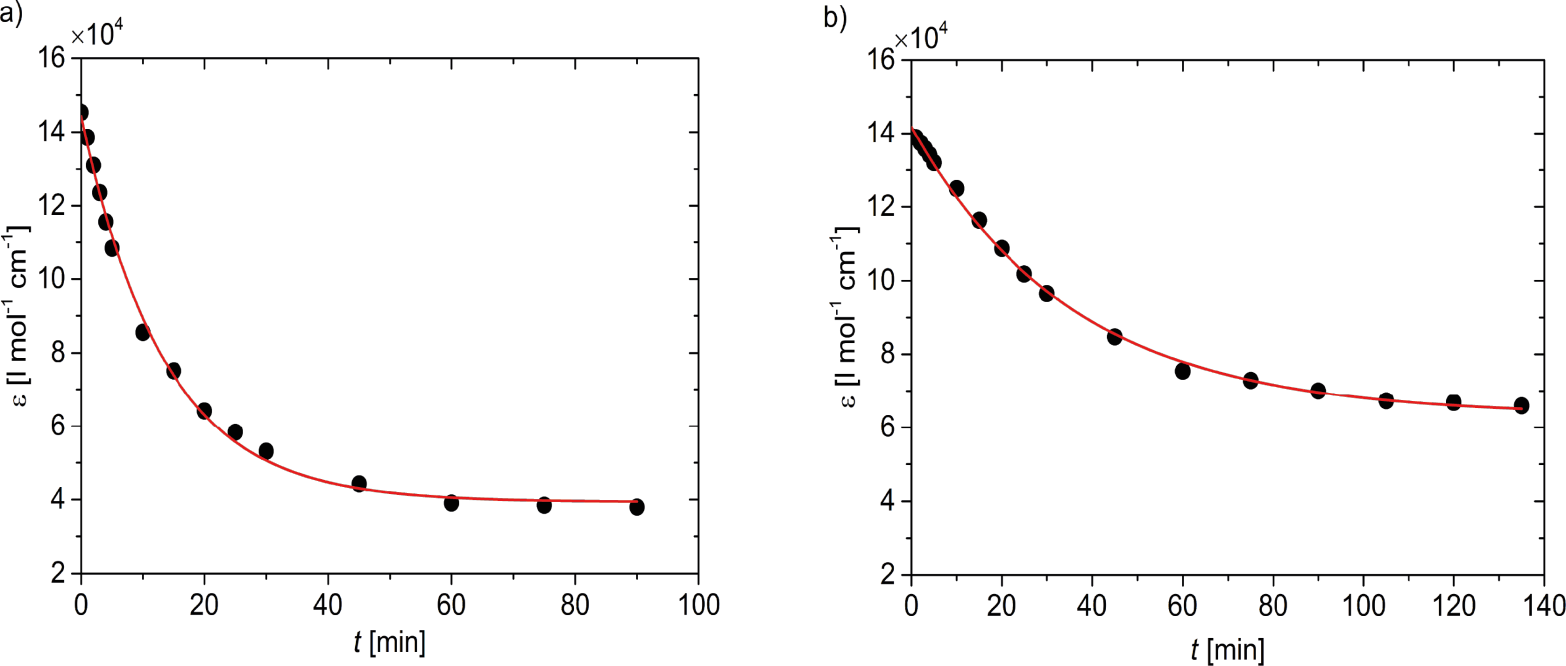
Continuous illumination of the trimer **1** in dichloromethane solution by UV light of 254 nm (a) and 365 nm (b). The exponential decay (red line) of the molar extinction coefficient illustrates the phototriggered dissociation of the parent structure.

Figure 3 displays the decay of the UV absorption of the trimer **1** in CH_2_Cl_2_ upon illumination by UV light at 254 nm and 365 nm respectively. The UV cuvette was exposed to a UV-lamp (8 W, 230 V, 50 Hz) at a constant distance of 1.5 cm. In order to avoid variations in concentrations, the solvent level in the cuvette was kept constant during the illumination experiment. An exponentially decaying molar extinction coefficient measured at the absorption maximum of 289 nm shows the photochemical activity of the compound. The initial extinction coefficient of ε_pc_ 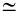 1.4 × 10^5^ L·mol^−1^·cm^−1^ corresponds to an absorption cross section of σ_pc_ 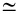 5.7 × 10^−16^ cm^2^. The photo-decay upon exposure to the more energetic 254 nm photons is about twice as fast compared to the 365 nm illumination.

In summary, the NMR measurements as well as the solution spectra display the expected photodegradation of these model compounds in solution. Their optical properties and photoreaction pathways may, however, vary in high vacuum, where the stabilization of the intermediates by surrounding solvent molecules is no longer possible and bathochromic line shifts and broadenings are common.

### Photoresponse of neutral model compounds in high vacuum

**Figure 4:**
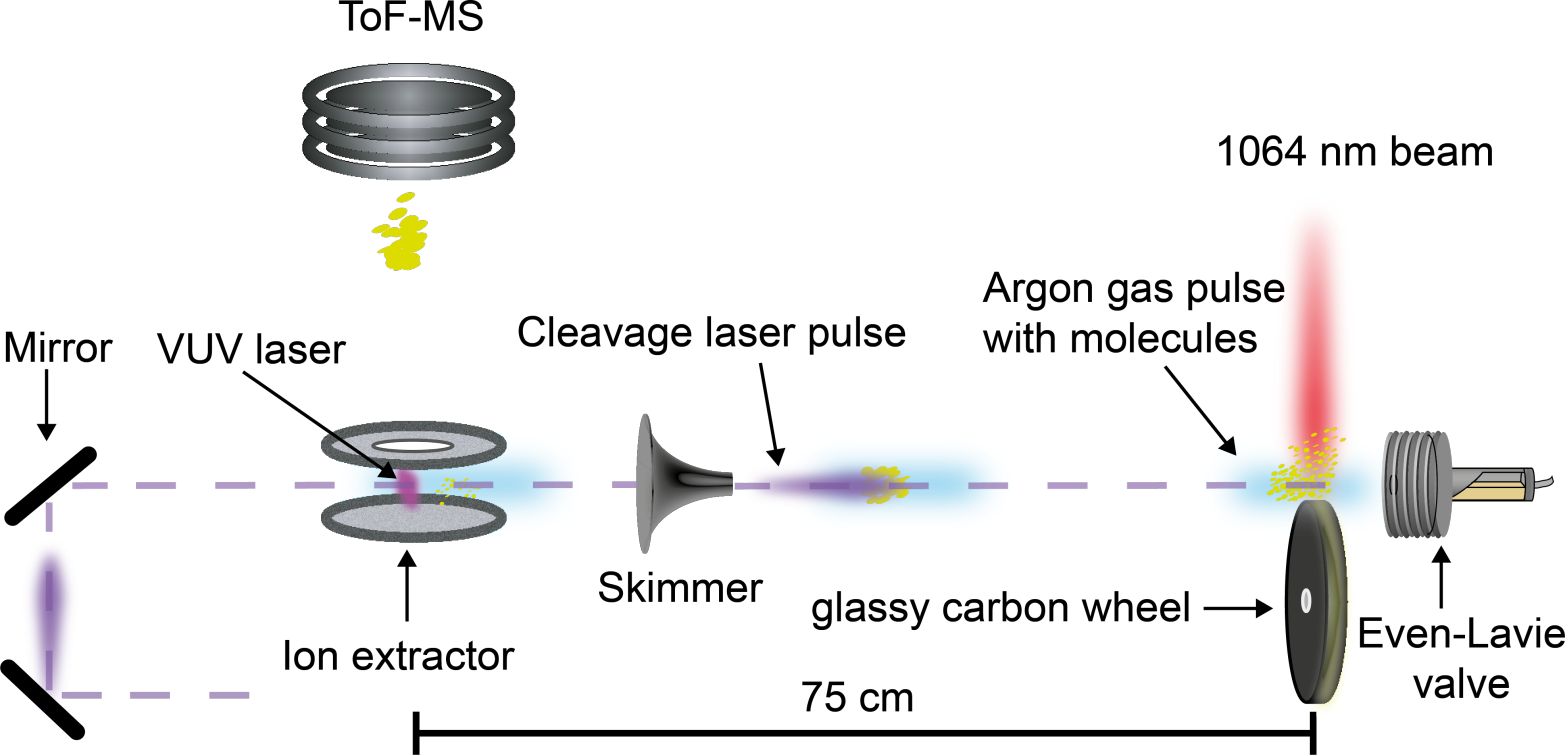
Molecular beam machine to study the photodepletion of the photoactive monomer in high vacuum. The pulsed infrared laser beam desorbs the molecules which are entrained by the adiabatically expanding neon gas jet. The neutral beam propagates for 75 cm in high vacuum before it is ionized by the pulsed radiation of a 157.6 nm F_2_ laser and analysed in a time-of-flight mass spectrometer. On the way to the detector the molecular beam is exposed to a collinear UV laser beam at 266 nm.

Experiments with neutral molecules were done in a molecular beam machine as sketched in Figure 4. In order to generate a pulsed beam of isolated neutral molecules we used nanosecond pulsed laser desorption into a supersonic neon seed gas jet [26]. The solid material was dissolved and spread on a glassy carbon wheel, from where it was desorbed by a 1064 nm laser pulse (10 ns duration, 38 mJ pulse energy, 10 Hz repetition rate, 3 mm beam waist). The laser pulse was synchronized with the opening of a pulsed Even–Lavie valve, 3 mm in front of the laser plume. Starting from a 30 bar stagnation pressure at room temperature, the neon gas cools down during the expansion and entrains the analyte molecules.

The molecular beam propagates over 75 cm towards the extraction region of a time-of-flight mass spectrometer where it is crossed and ionized by the beam of a pulsed vacuum-ultraviolet fluorine (F_2_) laser. The laser pulse has an energy of up to 1.1 mJ in 10 ns at a wavelength of 157.6 nm. The base pressure in the chamber was 6 × 10^−7^ mbar. The post-ionized particles were then extracted and the time-resolved ion signal was amplified and recorded by a fast digitizing oscilloscope. We verified that no ions were detected from the source in the absence of the VUV light.

In order to test for photoinduced dissociation in high vacuum, a 266 nm UV laser beam was aligned parallel and counter-propagating to the molecular beam. This UV beam was derived from a frequency quadrupled Nd–YAG laser with up to 5 mJ pulse energy, 7 ns pulse duration, and 10 Hz repetition rate (3.3(1) mm beam diameter).

In solution, the UV light led to strong cleavage of both the monomer **4** and the trimer **1** – with similar absorption cross sections at 254 nm and 366 nm – but faster decay at higher photon energy. Preliminary dissociation experiments at 355 nm did not reveal any major cleavage of the trimer **1** at the laser energies that were sufficient to cleave the monomer **4** at 266 nm. Because of this, the following experiments were performed exclusively on the monomer.

### Photoresponse of the monomer **4**

**Figure 5:**
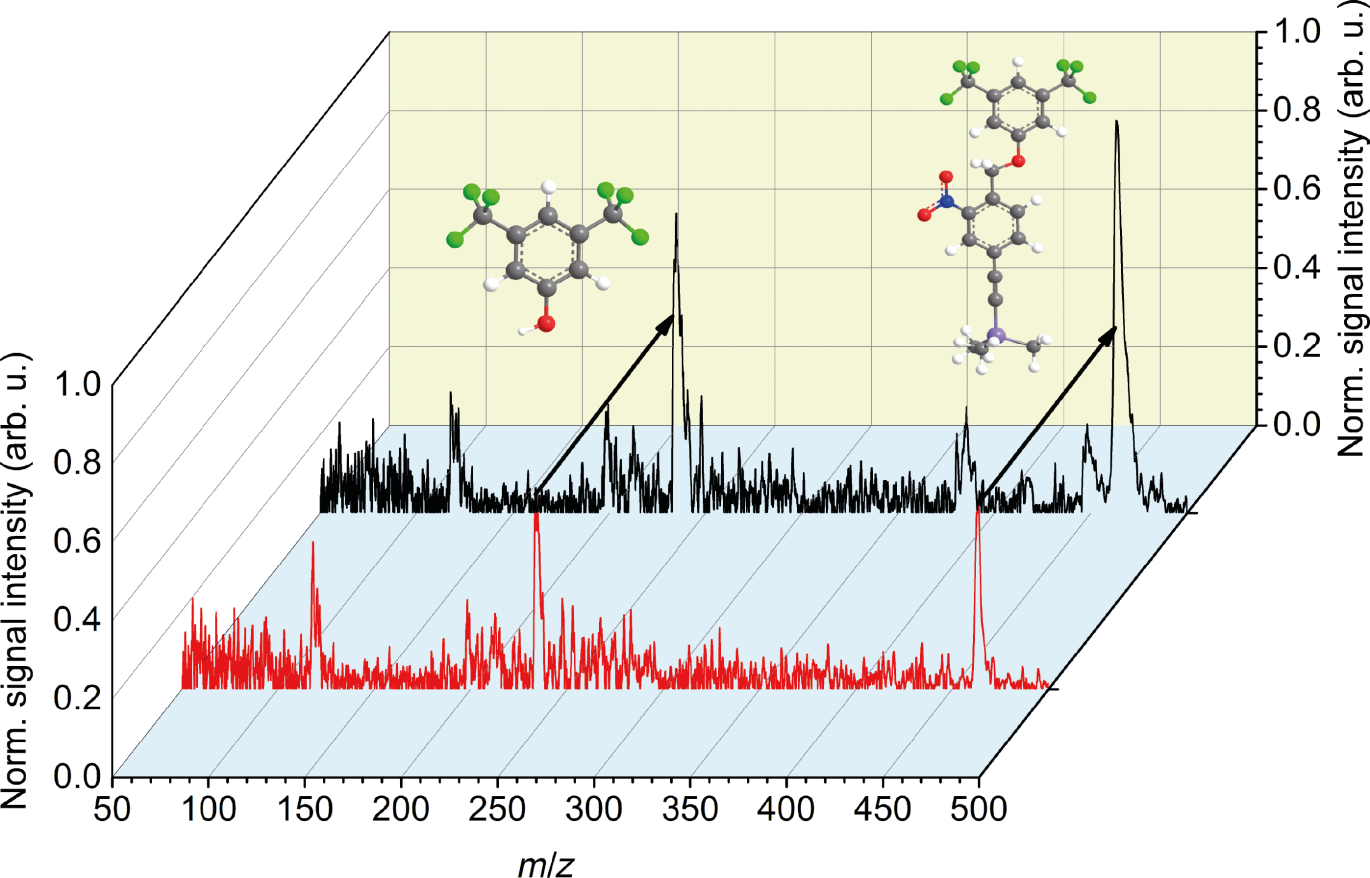
Photodepletion of the photocleavable nitrobenzyl derivative. When the dissociation laser pulse with an energy of 3.7(1) mJ in a beam diameter of 3.3(1) mm preceded the VUV ionization pulse by 600 µs, we observed beam depletion of the neutral parent molecule by a factor of four (red curve). The black spectrum represents the calibration curve without cleavage laser beam.

When we shine 266 nm light onto the propagating beam of isolated monomers, we observe a depletion of the parent signal, as shown in Figure 5 (red line). Even in the absence of any UV light, one can see fragments which we attribute to the IR laser desorption (black line in Figure 5). The strongest beam depletion was observed when the 266 nm laser pulse arrived about 600 µs before the VUV ionizing laser pulse, i.e., shortly behind the beam source. Neutral fragments emerging from that process will experience a dissociative recoil that kicks them beyond the detector acceptance angle of 4 mrad.

**Figure 6:**
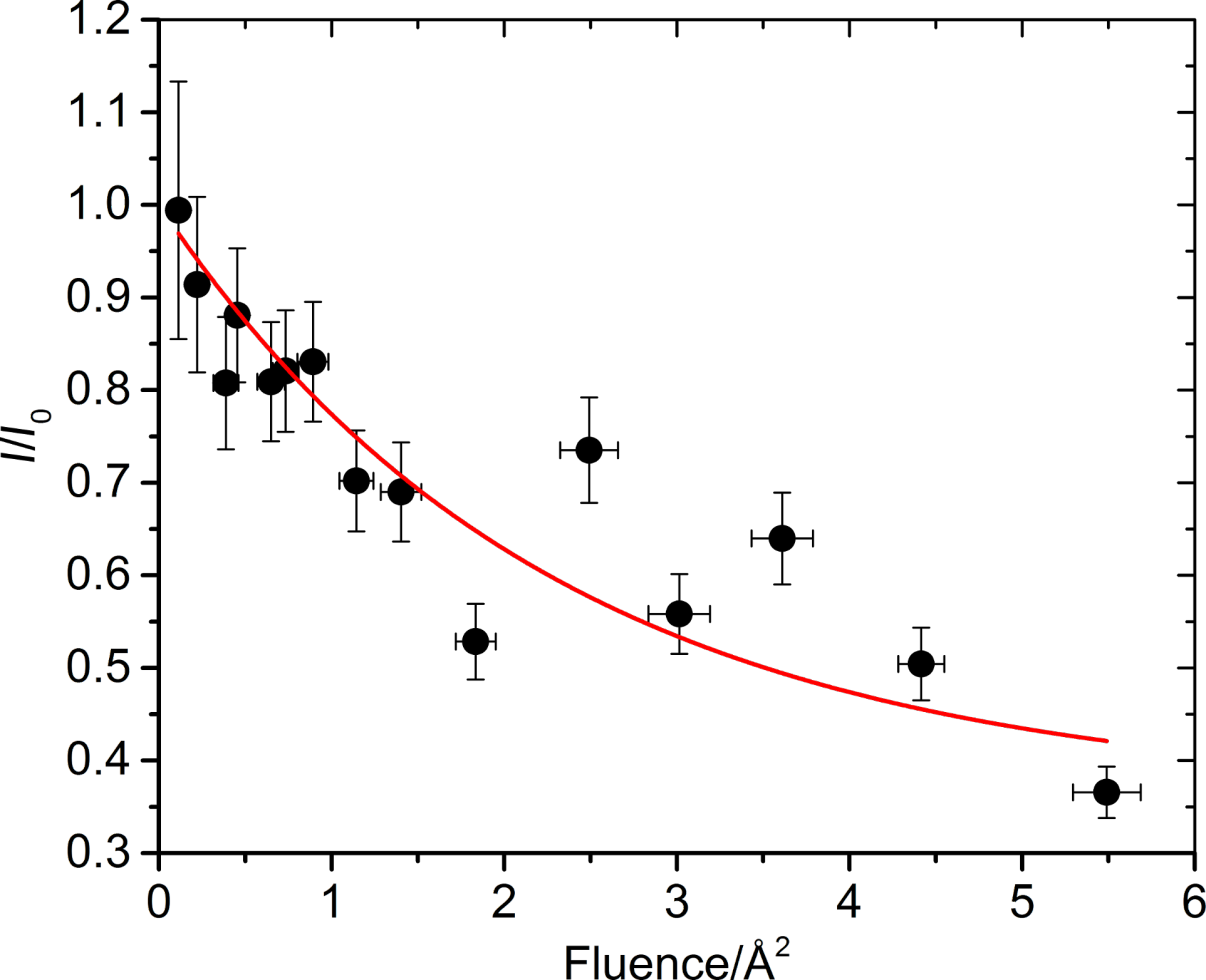
Depletion ratio, i.e., fraction of remaining parent molecules, versus laser fluence (photons per pulse and area). The continuous line is a fit to the data to derive the depletion cross section of the photoactive monomer.

In Figure 6, we plot the decay of the monomer ion signal as a function of the UV depletion pulse energy, with all other settings as for Figure 5. We deplete the beam by more than 50%. Using the exponential decay


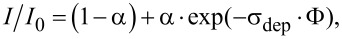


we find the experimental beam overlap parameter α = 0.64(8) and an in-vacuum beam depletion cross section σ_dep_ = 0.4(2) A^2^ = 4(2) × 10^−17^ cm^2^ where Φ is the laser fluence, i.e., the photon number in a single pulse per laser beam area.

The depletion of the parent signal is attributed to photoactivated dissociation. To exclude the contribution of ionization, we have verified that the 266 nm light does not lead to any additional photoions when the UV laser pulse coincides with the ionizing VUV pulse. In this configuration it only enhances the parent signal, indicating absorption and heating of the monomer. We cannot exclude that the same thermal process leads to delayed fragmentation and subsequent beam depletion. However, the strong decay of the parent peak compared to its fragment is consistent with the picture that the fragment has no second remaining ‘weak link’.

## Discussion and Conclusion

A novel concept for the realization of optical gratings based on photocleavable subunits is presented. For that purpose, we have designed, synthesized and characterized a molecular tag based on a 2-(phenoxymethyl)-1-nitrobenzene motive comprising an ethynyl group enabling its modular attachment by covalent coupling chemistry. The covalent attachment has been demonstrated with the trimeric model compound **1** while the here presented preliminary gas-phase photocleavage experiments have been performed with the monomeric building block **4**, which displayed favourable photodissociation features compared to **1**. Interestingly we find that photoactivated molecular beam depletion is even possible in high vacuum and with a cross section high enough for accessible laser sources.

When isolated in high vacuum many details of the photochemistry are expected to change, opening interesting questions for future research with regard to the time-dependent reaction pathways in the gas-phase.

**Figure 7:**
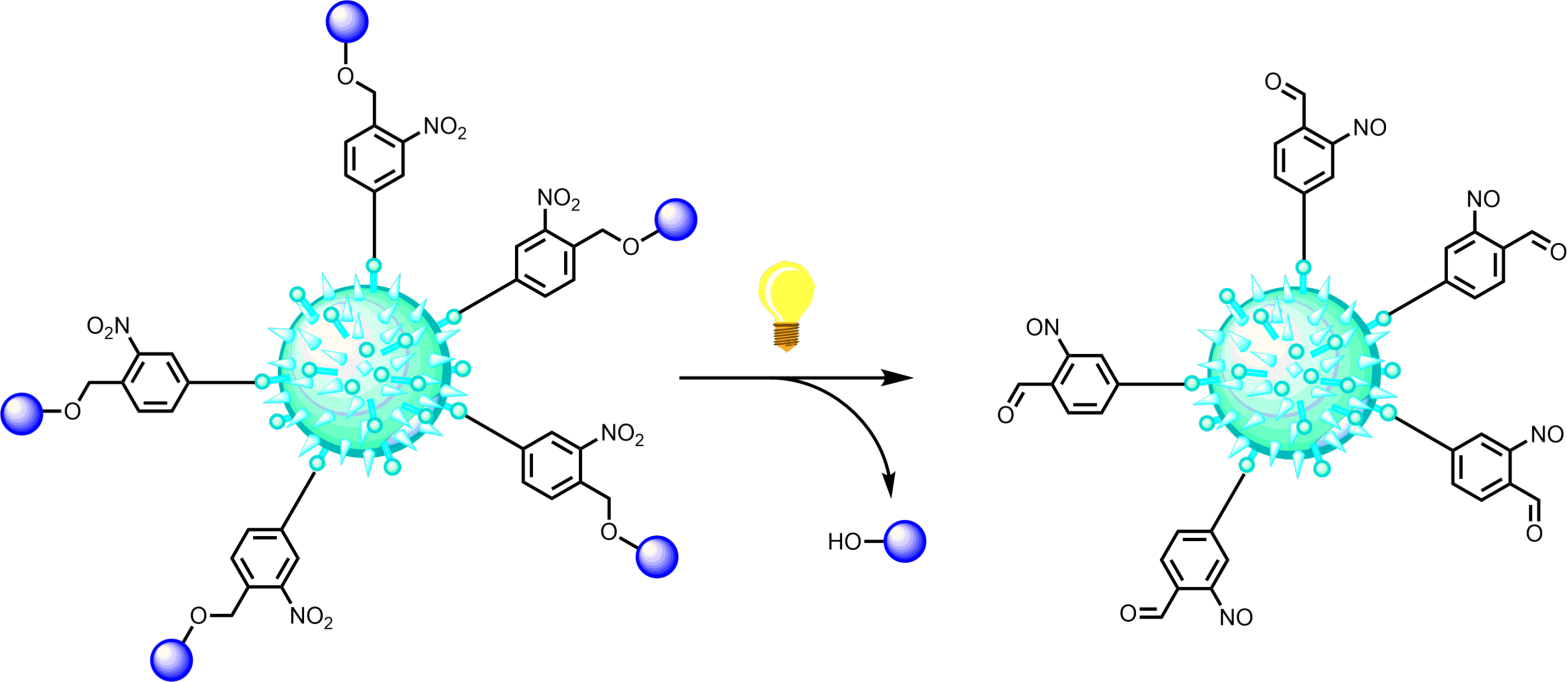
Bond-selective dissociation and photoinduced beam depletion shall enable novel absorptive optical gratings for complex nanobiological materials, which cannot be handled by established optical manipulation techniques based on photoionization. The idea is here illustrated for a nitrobenzyl tagged virus (artist’s view).

For future applications in molecule interferometry with complex biomolecules, it will be important that beam depletion is possible at a wavelength which is not absorbed by the untagged molecule. Otherwise the photon would be dissipated without triggering any action. Our labels are chosen such that they respond at 250–270 nm and 350–370 nm, i.e., at wavelengths longer than typical resonance lines in non-aromatic biomolecules. We expect photocleavage to become important for the realization of optical gratings and for the photoactivated change of the charge state in mesoscopic objects. Both will be crucial for molecular quantum optics and future matter-wave experiments. An open future challenge will be to explore how well this method scales with the size of particles from individual monomer to trimers, chromophore-labelled biomolecules up to decorated viruses, as displayed as visionary sketch in Figure 7. Currently we are investigating the potential of the concept for biomolecules with tagged oligo- and poly-peptides.

## Supporting Information

Here we describe the synthesis and characterisation of the chemical compounds and the photocleavable molecules used for this study.

File 1 Synthetic protocols.

## Dedication

This contribution is dedicated to Herbert Gleiter, a pioneer of nanotechnology with a joint passion in the foundations of physics and matter-waves.

## References

[1] Arndt M, Nairz O, Voss-Andreae J, Keller C, van der Zouw G, Zeilinger A (1999). Nature.

[2] Juffmann T (2012). Surface based detection schemes for molecular matter-wave interferometry.

[3] Tino G, Kasevich M (2014). Atom Interferometry. Proceedings of the International School of Physics "Enrico Fermi".

[4] Arndt M, Hornberger K (2014). Nat Phys.

[5] Arndt M, Juffmann T, Vedral V (2009). HFSP J.

[6] Arndt M, Dörre N, Eibenberger S, Haslinger P, Rodewald J, Hornberger K, Nimmrichter S, Mayor M, Tino G M, Kasevich M (2014). Matter-wave interferometry with composite quantum objects. Atom Interferometry, Proceedings of the International School of Physics "Enrico Fermi".

[7] Arndt M (2014). Phys Today.

[8] Gerlich S, Gring M, Ulbricht H, Hornberger K, Tüxen J, Mayor M, Arndt M (2008). Angew Chem, Int Ed.

[9] Juffmann T, Milic A, Müllneritsch M, Asenbaum P, Tsukernik A, Tüxen J, Mayor M, Cheshnovsky O, Arndt M (2012). Nat Nanotechnol.

[10] Gerlich S, Eibenberger S, Tomandl M, Nimmrichter S, Hornberger K, Fagan P J, Tüxen J, Mayor M, Arndt M (2011). Nat Commun.

[11] Gerlich S, Hackermüller L, Hornberger K, Stibor A, Ulbricht H, Gring M, Goldfarb F, Savas T, Müri M, Mayor M (2007). Nat Phys.

[12] Hornberger K, Gerlich S, Haslinger P, Nimmrichter S, Arndt M (2012). Rev Mod Phys.

[13] Haslinger P, Dörre N, Geyer P, Rodewald J, Nimmrichter S, Arndt M (2013). Nat Phys.

[14] Brezger B, Arndt M, Zeilinger A (2003). J Opt B: Quantum Semiclassical Opt.

[15] Brezger B, Hackermüller L, Uttenthaler S, Petschinka J, Arndt M, Zeilinger A (2002). Phys Rev Lett.

[16] Hackermüller L, Uttenthaler S, Hornberger K, Reiger E, Brezger B, Zeilinger A, Arndt M (2003). Phys Rev Lett.

[17] Eibenberger S, Gerlich S, Arndt M, Mayor M, Tüxen J (2013). Phys Chem Chem Phys.

[18] Reiger E, Hackermüller L, Berninger M, Arndt M (2006). Opt Commun.

[19] Haslinger P (2013). A universal matter-wave interferometer with optical gratings.

[20] Dörre N, Rodewald J, Geyer P, von Issendorff B, Haslinger P, Arndt M (2014). Phys Rev Lett.

[21] Schlag E W, Grotemeyer J, Levine R D (1992). Chem Phys Lett.

[22] Klán P, Šolomek T, Bochet C G, Blanc A, Givens R, Rubina M, Popik V, Kostikov A, Wirz J (2013). Chem Rev.

[23] Sezer U, Schmid P, Felix L, Mayor M, Arndt M (2015). J Mass Spectrom.

[24] Sezer U, Wörner L, Horak J, Felix L, Tüxen J, Götz C, Vaziri A, Mayor M, Arndt M (2015). Anal Chem.

[25] Il'ichev Y V, Wirz J (2000). J Phys Chem A.

[26] Geyer P, Sezer U, Rodewald J, Mairhofer L, Dörre N, Haslinger P, Eibenberger S, Brand C, Arndt M (2016). Phys Scr.

