# Supplementary material for: Selective photodissociation of tailored molecular tags as a tool for quantum optics
Source: Beilstein J Nanotechnol. 2017 Feb 2;8:325–33. doi: 10.3762/bjnano.8.35 (PMC5301912; doi:10.3762/bjnano.8.35)
Supplement: File 1 — Synthetic protocols. [file Beilstein_J_Nanotechnol-08-325-s001.pdf]

Supporting Information

for

Selective photodissociation of tailored molecular tags as a tool for quantum optics

Ugur Sezer[1], Philipp Geyer[1], Moritz Kriegleder[1], Maxime Debiossac[1], Armin Shayeghi[1], and Markus Arndt[1], Lukas Felix[2] and Marcel Mayor[2,3,4*]

Address: [1]Faculty of Physics, VCQ, University of Vienna, Boltzmanngasse 5, A-1090 Vienna, Austria, [2]Department of Chemistry, University of Basel, St. Johannsring 19, CH-4056 Basel, Switzerland, [3]Institute of Nanotechnology (INT), Karlsruhe Institute of Technology (KIT), Hermann-von-Helmholtz-Platz 1, D-76344 Eggenstein-Leopoldshafen, Germany and [4]Lehn Institute of Functional Materials (LIFM), Sun Yat-Sen University (SYSU), Xingang Rd. W., Guangzhou, China

Email: Marcel Mayor - marcel.mayor@unibas.ch

* Corresponding author

Synthetic protocols

General remarks:

Reagents and solvents: All commercially available starting materials were of reagent grade and used as received from Fluka AG (Buchs, Switzerland), Acros AG (Basel, Switzerland), Merck (Darmstadt, Germany), Alfa Aesar (Karlsruhe, Germany), ABCR (Karlsruhe, Germany), Aldrich (Buchs, Switzerland), Fluorochem (Hadfield,

United Kingdom) and Porphyrin Systems GbR (Norderstedt, Germany). The solvents for chromatography, crystallization and for extraction were used in technical grade. The solvent for HPLC analyses and GPC was of HPLC grade. Dry THF, dry DMF and dry CH_2Cl_2 were purchased from Acros, stored over 4 Å molecular sieves and handled under argon. For an inert atmosphere, Argon 4.8 from PanGas AG (Dagmersellen, Switzerland) was used.

Syntheses: All reactions with reagents that are sensitive to air or moisture were performed under an argon atmosphere using Schlenk technique, only dry solvents were used and the glassware was heated out before use.

Characterization:

^{1}H Nuclear Magnetic Resonance (^{1}H NMR): Bruker DRX-NMR (600 MHz or 500 MHz), Bruker DPX-NMR (400 MHz) and Bruker BZH-NMR (250 MHz) instruments were used to record the spectra. Chemical shifts (δ) are reported in parts per million (ppm) relative to residual solvent peaks ($CDCl_3$: 7.26 ppm) or trimethylsilane (TMS: 0.00 ppm), and coupling constants (J) are reported in Hertz (Hz). The bond distance of the coupling constant is stated with a superscript number (^nJ). NMR solvents were obtained from Cambridge Isotope Laboratories, Inc. (Andover, MA, USA). The measurements were done at room temperature. The multiplicities are written as s = singlet, d = doublet, t = triplet, q = quartet, quin = quintet, m = multiplet and brs = broad.

^{13}C Nuclear Magnetic Resonance (^{13}C NMR): Bruker DRX-NMR (126 MHz) and Bruker DPX-NMR (101 MHz) instruments were used to record the spectra. Chemical shifts (δ) are reported in parts per million (ppm) relative to residual solvent peaks ($CDCl_3$: 77.0 ppm or TMS: 0.0 ppm). The measurements were done at room

temperature. The multiplicities are written as s = singlet, d = doublet, t = triplet, q = quartet, quin = quintet and m = multiplet. The coupling constants (J) are reported in Hertz (Hz) and are just stated in fluorine containing molecules. The signals of fluoroalkylsulfanyl segments were not assigned due to the coupling to fluorine.

^{19}F Nuclear Magnetic Resonance (^{19}F NMR): A Bruker DPX-NMR (377 MHz) instrument was used to record the spectra. Chemical shifts (δ) are reported in parts per million (ppm), uncorrected. The measurements were done at room temperature. Coupling constants (J) are reported in Hertz (Hz). The multiplicities are written as: s = singlet, d = doublet, t = triplet, q = quartet, quin. = quintet, m = multiplet.

Mass Spectrometry (MS): Matrix-Assisted Laser Desorption Ionization Time of Flight (MALDI-TOF) mass spectra were performed with a Bruker Microflex LRF. MALDI-TOF spectra were calibrated using CsI_3 cluster.[1] DCTB (*trans*-2-[3-(4-*tert*-Butylphenyl)-2-methyl-2-propenylidene]malononitrile) was used as matrix if needed.[2] High-resolution mass spectra were taken on a Bruker solariX (ESI/MALDI-FTICR-MS), Bruker maXis 4G (QTOF-ESI).

***(4-Bromo-2-nitrophenyl)methanol* (2)[3]:** In an oven dried flask 1-(4-bromo-2-nitrophenyl)ethan-1-one, 10 g, 43.5 mmol, 1.0 equiv) was dissolved in dry THF (140 mL) and $NaBH_4$ (3.34 g, 84.7 mmol, 1.95 equiv) was added in portions. The reaction was stirred for 15 min at room temperature. When TLC showed full conversion the reaction mixture was cooled down to 0 °C and aq. NH_4Cl (20 %, 100 ml) was slowly added. After extraction with TBME combined organic phases

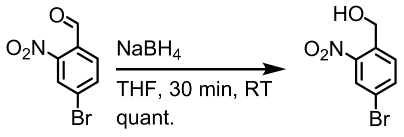

2

[1] Lou, X.; van Dongen, J. L. J.; Meijer, E. W. *J. Am. Soc. Mass Spectrom.* **2010**, *21* (7), 1223–1226.
[2] Winter, J. D.; Deshayes, G.; Boon, F.; Coulembier, O.; Dubois, P.; Gerbaux, P. *J. Mass Spectrom.* **2011**, *46* (3), 237–246.
[3] Lemasson, F.; Tittmann, J.; Hennrich, F.; Stürzl, N.; Malik, S.; Kappes, M. M.; Mayor, M. *Chem. Commun.* **2011**, *47* (26), 7428–7430.

were washed with water and brine. After drying over anhydrous $MgSO_4$ the solvent was removed under reduce pressure. The remaining residue was purified by column chromatography (silica gel, cyclohexane/ethyl acetate; 2:1) to give (4-bromo-2-nitrophenyl)methanol (**2**) as a colorless solid in quantitative yield (10.01 g, 43.5 mmol, 230.95 g/mol). **^{1}H-NMR** (400 MHz, $CDCl_3$, δ/ppm): 2.45 (brs, 1 H, OH), 4.96 (s, 2 H, CH_2), 7.67 (d, $^3J_{HH}$ = 8.3 Hz, 1 H, ArH), 7.80 (dd, $^3J_{HH}$ = 8.3 Hz, $^4J_{HH}$ = 2.0 Hz, 1 H, ArH), 8.25 (d, $^4J_{HH}$ = 2.0 Hz, 1 H, ArH); **^{13}C-NMR** (101 MHz, $CDCl_3$, δ/ppm): 62.12 (s, 2C), 121.65, 128.01, 131.23, 136.03, 137.18, 147.92; All other analytical data were according to literature values.

***1-((3,5-Bis(trifluoromethyl)phenoxy)methyl)-4-bromo-2-nitrobenzene* (3):** The whole reaction was performed in the absence of light. A solution of 4-bromo-2-nitrophenyl)methanol (**2**, 4 g, 17.24 mmol, 1.0 equiv), 3,5-bis(trifluoromethyl)phenol (4.88 g, 20.6 mmol, 1.2 equiv) and triphenylphosphine (5.26 g, 20.6 mmol, 1.2 equiv) in dry THF (125 mL) was cooled to 0 °C. DIAD (4.3 mL, 20.6 mmol, 1.2 equiv) was added dropwise within 15 min. The reaction was allowed to warm to room temperature and stirred for 12 h. When TLC showed full conversion of the starting materials the solvent was removed in vacuo and the remaining residue purified by column chromatography (silica gel, cylcohexane/ethyl acetate; 10:1) yielding 1-((3,5-bis(trifluoromethyl)phenoxy)methyl)-4-bromo-2-nitrobenzene (**3**) as a colorless solid in 96% yield (7.32 g, 16.48 mmol, 442.96 g/mol). **^{1}H-NMR** (400 MHz, $CDCl_3$, δ/ppm): 5.51 (s, 2 H, CH_2), 7.24 (s, 2 H, ArH), 7.55 (s, 1 H, ArH), 7.77 (d, $^3J_{HH}$ = 8.4 Hz, 1 H, ArH), 7.86 (dd, $^3J_{HH}$ = 8.4 Hz, $^4J_{HH}$ = 2.0 Hz, 1 H, ArH), 8.36 (d, $^4J_{HH}$ = 2.0 Hz, 1 H, ArH); **^{19}F-NMR** (376 MHz, $CDCl_3$, δ/ppm): -63.0 (s, 6 F, 2 x CF_3); **^{13}C-NMR** (101 MHz, $CDCl_3$, δ/ppm): 67.12 (s),

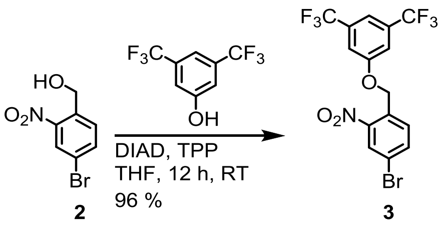

115.20 (m), 115.46 (m), 121.62, 122.40, 124.33, 128.29, 129.97, 131.01, 133.19 (m), 137.21, 147.27, 158.42; **HRMS** (ESI): *m/z* calcd for $C_{15}H_8BrF_6NO_3$: 442.9592; found 441.9591.

***((4-((3,5-Bis(trifluoromethyl)phenoxy)methyl)-3-nitrophenyl)ethynyl)trimethylsilane* (4):** In an oven dried two neck flask 4-bromo-1-((4-fluoro-phenoxy)methyl)-2-nitrobenzene (**3**, 100 mg, 0.226 mmol, 1.0 equiv) was dissolved in triethylamine (6 mL). The solution was degassed for 20 min in an argon stream. Then copper(I) iodide (0.44 mg, 1 mol %), and tetrakis(triphenylphosphine)palladium (1.32 mg, 0.5 mol %) were added, and the resulting solution degassed again for 10 min in an argon stream. Trimethylsilylacetylene (0.05 ml, 0.339 mmol, 1.5 equiv) was added and the reaction heated to reflux for 12 h. After filtration through Celite® the solution was dissolved in TBME, and combined organic phases washed with water and brine. After drying over anhydrous Na_2SO_4 the solvent was removed under reduced pressure. The crude was purified by column chromatography (silica gel, cyclohexane/ethyl acetate; 5:1) to obtain ((4-((3,5-Bis(trifluoromethyl)phenoxy)methyl)-3-nitrophenyl)ethynyl)-trimethyl-silane (**4**) as a colorless solid in 99% yield (103 mg, 0.223 mmol, 461.09 g/mol). **^{1}H-NMR** (400 MHz, $CDCl_3$, δ/ppm): 0.27 (s, 9 H, TMS), 5.56 (s, 2 H, CH_2), 7.46 (s, 2 H, ArH), 7.56 (s, 1 H, ArH), 7.77 (dd, $^3J_{HH}$ = 8.1 Hz, $^4J_{HH}$ = 1.6 Hz, 1 H, ArH), 7.82 (d, $^3J_{HH}$ = 8.1 Hz, 1 H, ArH), 8.26 (d, $^4J_{HH}$ = 1.6 Hz, 1 H, ArH); **^{19}F-NMR** (376 MHz, $CDCl_3$, δ/ppm): -63.3 (s, 6 F, ArF); **^{13}C-NMR** (101 MHz, CD_2Cl_2, δ/ppm): -0.08 (s, TMS), 68.05 (s), 98.70 (s), 102.08 (s), 115.80 (m), 115.94 (m), 122.38 (s), 125.10 (s), 128.86 (s), 129.24 (s), 132.46 (s), 133.45 (q), 137.49 (s), 147.36 (s), 159.26 (s); **HRMS** (ESI): *m/z* calcd for $C_{20}H_{17}F_6NO_3Si$: 461.0882 found 460.0809.

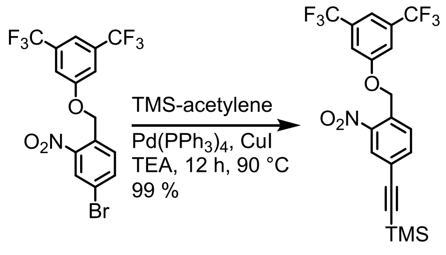

3
4

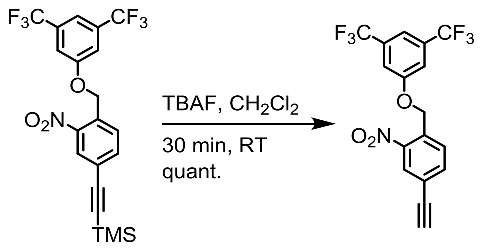

4 **5**

***1-((3,5-Bis(trifluoromethyl)phenoxy)methyl)-4-ethynyl-2-nitrobenzene* (5):** ((4-((3,5-Bis-(trifluoromethyl)phenoxy)methyl)-3-nitrophenyl)-ethynyl)-trimethylsilane (**4**, 100 mg, 0.226 mmol, 1.0 equiv) was dissolved in dichloromethane (150 mL) under an atmosphere of argon. TBAF (1 M in THF, 11.9 mL, 11.9 mmol, 2.5 equiv) was added, and the solution stirred for 15 min at room temperature. When TLC showed full conversion of the starting material methanol (100 mL) was added. After evaporation of the solvents the crude was purified by column chromatography (silica gel, cyclohexane/ethyl acetate; 10:1) to obtain 1-((3,5bis(trifluoromethyl)-phenoxy)methyl)-4-ethynyl-2-nitrobenzene (**5**) in quantitative yield (1.85 g, 4.77 mmol, 389.05 g/mol). **^{1}H-NMR** (400 MHz, $CDCl_3$, δ/ppm): 3.16 (s, 1 H, CH), 5.56 (s, 2 H, CH_2), 7.43 (s, 2 H, ArH), 7.54 (s, 1 H, ArH), 7.81 (dd, $^{3}J_{HH}$ = 8.1 Hz, $^{4}J_{HH}$ = 1.6 Hz, 1 H, ArH), 7.86 (d, $^{3}J_{HH}$ = 8.1 Hz, 1 H, ArH), 8.31 (d, $^{4}J_{HH}$ = 1.6 Hz, 1 H, ArH); **^{19}F-NMR** (376 MHz, $CDCl_3$, δ/ppm): -63.0 (s, 6 F, ArF); **^{13}C-NMR** (101 MHz, $CDCl_3$, δ/ppm): 67.43 (s), 80.68 (s), 80.73 (s), 115.38 (m), 115.55 (m), 123.14 (q), 123.76 (s), 128.74 (s), 128.80 (s), 132.50 (s), 133.33 (q), 137.39 (s), 146.38 (s), 158.62 (s); **HRMS** (ESI): *m/z* calcd for $C_{17}H_9F_6NO_3Si$: 389.0487 found 388.0417.

***1,3,5-Tris((4-((3,5-bis(trifluoromethyl)phenoxy)methyl)-3-nitrophenyl)ethynyl)-benzene* (1):**

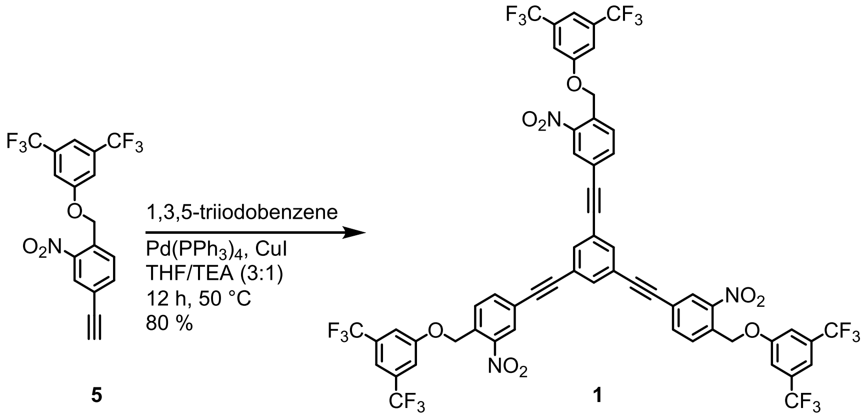

1,3,5-Triiodobenzene (65.2 mg, 0.14 mmol, 1.0 equiv) was dissolved in THF/TEA (3:1, 20 mL) and degassed for 20 min in a constant argon stream. Copper(I) iodide (1.4 mg, 5 mol %) and tetrakis(triphenylphosphine)palladium(0) (8.4 mg, 5 mol %) were added and the solution degassed again for 10 min in a constant argon stream. 1-((3,5-Bis(trifluoromethyl)phenoxy)methyl)-4-ethynyl-2-nitrobenzene (**5**, 250 mg, 0.64 mmol, 4.5 equiv) was added and the reaction mixture heated to 50 °C for 12 h. The completion of the reaction was checked by TLC. When all starting material was consumed the reaction mixture was cooled to room temperature and extracted with TBME. Combined organic phases were washed with water and brine, dried over anhydrous Na_2SO_4, and the solvent removed under reduced pressure. The crude was recrystallized from chloroform to obtain 1,3,5-tris((4-((3,5-bis(trifluoromethyl)-phenoxy)methyl)-3-nitrophenyl)ethynyl)benzene (**1**) as a colorless solid in 80 % yield (142 mg, 1.54 mmol, 1239.15 g/mol). **^{1}H-NMR** (400 MHz, THF-*d_8*, δ/ppm): 5.72 (s, 6 H, CH_2), 7.67 (m, 9 H, ArH), 7.94 (s, 3 H, ArH), 7.94 (m, 6 H, ArH), 8.38 (m, 2 H, ArH); **^{19}F-NMR** (376 MHz, THF-*d_8*, δ/ppm): -63.7 (s, 18 F, ArF); **^{13}C-NMR** (101 MHz, THF-*d_8*, δ/ppm): 68.61 (s), 89.29 (s), 90.52 (s), 115.86 (m), 116.77 (s), 123.15 (s), 124.54 (s), 124.79 (s), 125.86 (s), 128.87 (s), 130.14 (s), 134.04 (q), 135.90 (s), 137.26 (s), 148.71 (s); **HRMS** (MALDI/ESI): *m/z* calcd for $C_{57}H_{27}F_{18}N_3NaO_9$: 1262.1352 found 1262.1370.
